# Pharmacokinetics, tissue distribution and mass balance of radiolabeled dihydroartemisinin in male rats

**DOI:** 10.1186/1475-2875-8-112

**Published:** 2009-05-26

**Authors:** Lisa H Xie, Qigui Li, Jing Zhang, Peter J Weina

**Affiliations:** 1 Department of Pharmacology, Division of Experimental Therapeutics, Walter Reed Army Institute of Research, 503 Robert Grant Avenue, Silver Spring, Maryland 20910-7500, USA

## Abstract

**Background:**

Dihydroartemisinin (DHA), a powerful anti-malarial drug, has been used as monotherapy and artemisinin-based combination therapy (ACT) for more than decades. So far, however, the tissue distribution and metabolic profile of DHA data are not available from animal and humans.

**Methods:**

Pharmacokinetics, tissue distribution, mass balance, and elimination of [^14^C] DHA have been studieded in rats following a single intravenous administration. Protein binding was performed with rat and human plasma. Drug concentrations were obtained up to 192 hr from measurements of total radioactivity and drug concentration to determine the contribution by the parent and metabolites to the total dose of drug injected from whole blood, plasma, urine and faecal samples.

**Results:**

Drug was widely distributed after 1 hr and rapidly declined at 24 hr in all tissues except spleen until 96 hrs. Only 0.81% of the total radioactivity was detected in rat brain tissue. DHA revealed a high binding capacity with both rat and human plasma proteins (76–82%). The concentration of total radioactivity in the plasma fraction was less than 25% of that in blood total. Metabolism of DHA was observed with high excretion via bile into intestines and approximately 89–95% dose of all conjugations were accounted for in blood, urine and faeces. However, the majority of elimination of [^14^C] DHA was through urinary excretion (52% dose). The mean terminal half-lives of plasma and blood radioactivity (75.57–122.13 h) were significantly prolonged compared with that of unchanged DHA (1.03 h).

**Conclusion:**

In rat brain, the total concentration of [^14^C] was 2-fold higher than that in plasma, indicating the radioactivity could easily penetrate the brain-blood barrier. Total radioactivity distributed in RBC was about three- to four-fold higher than that in plasma, suggesting that the powerful anti-malarial potency of DHA in the treatment of blood stage malaria may relate to the high RBC binding. Biliary excretion and multiple concentration peaks of DHA have been demonstrated with high urinary excretion due to a most likely drug re-absorption in the intestines (enterohepatic circulation). The long lasting metabolites of DHA (> 192 hr) in the rats may be also related to the enterohepatic circulation.

## Background

Dihydroartemisinin (DHA) is the active metabolite of the more widely used artesunate and artemether. DHA is manufactured as an oral anti-malarial drug used in southern Asia and Africa. Recently, Walter Reed Army Institute of research (WRAIR) (explicit abbreviation) has chosen artesunate (AS) injection as the blood stage compound for severe and complicated malaria and it has been demonstrated that AS is a pro-drug of DHA [1], which has same efficacy to AS and is three to five times more active than the other artemisinin derivatives [2-5]. Artesunate is a more potent anti-malarial drug than arteether and artemether, which may be explained by the conversion to DHA with the DHA/AS ratios of 0.31–2.74 in animal species [6,7], and 2.81–9.71 in malaria humans [8,9]. A study demonstrated that only DHA was rapidly effective against all stages of the parasite life cycle and completely inhibited the parasite growth within the shortest exposure time when compared to all other artemisinin drugs [10]. It is also felt by many clinicians that AS administered intravenously is the most effective for treatment of severe malaria due to the high DHA levels [11].

Cotecxin^® ^(DHA tablet) has been used in China and Thailand since 1991 [12]. Given alone, however, this artemisinin derivative must be given daily for seven days for optimum cure rates. Five-day regimens give lower cure rates and three-day regimens of artemisinin monotherapy are associated with very high failure rates (40–80%) in malaria patients [13]. It is widely accepted that artemisinin-based combination therapy (ACT) provides the best available treatment for uncomplicated multidrug-resistant falciparum malaria [14,15]. ACT gives reliably rapid therapeutic responses, provides high cure rates with three-day treatment regimens, is well tolerated, reduces gametocyte carriage and provides mutual protection for the partner drugs against the emergence of resistance [16]. DHA-piperaquine (Artekin^®^) has a cost advantage over other combinations (US$ 1 for an adult treatment) making it a potential best candidate for deployment in Vietnam, Southeast Asia and Africa [17].

Although the use of DHA has spread rapidly and pharmacokinetic research in animals and humans has been done, basic questions regarding tissue distribution, protein binding, and metabolic profile of DHA, especially following intravenous treatment, have not been answered. The tissue distribution investigation of radiolabeled AS had been conducted by Li et al. [18]. Radioactivity derived from [^14^C]-AS was eliminated from plasma in three phases with half-lives of 0.12, 13.2, and 76.2 hours. Unchanged AS was eliminated from plasma in two apparent phases with half-lives of 0.14 and 0.43 hours, suggesting that the total radioactivity of AS lasted much longer than unchanged AS in rats. Through 192 hours after injection, approximately 56%, 38%, and 1.6% of the dose was eliminated via urine, faeces, and cage rinses, respectively. Unchanged AS was eliminated within one hour, indicating long-lasting radioactivity may be the result of AS metabolites including DHA [18].

However, there is no information on the disposition and excretion of DHA after intravenous injection of its parent drug, AS. Also, previous papers did not discriminate the metabolic profiles of AS and DHA. The present study was undertaken to ascertain the pharmacokinetics, tissue distribution, mass balance, and elimination of radiolabeled DHA in rats and protein binding in both of rat and human plasma. In accordance with FDA guidance [19], any parent compound and its major metabolite(s) that achieve, or are expected to achieve, systemic exposure in human should be evaluated in pharmacology studies prior to human clinical trials [19]. In the present study, DHA was administered intravenously in male rats for pharmacokinetics, tissue distribution, protein binding, and metabolic profile evaluations.

## Methods

### Chemicals

The test unlabeled compound used in this study is dihydroartemisinin (10'dihydro-artemisinin, DHA), which was prepared as a 25% cremophore/0.9% saline solution. Unlabeled DHA was synthesized and manufactured by Knoll AG (Switzerland) and repackaged by BASF Pharmaceuticals (Ludwigshafen, Germany). It was supplied through the Division of Experimental Therapeutics at Walter Reed Army Institute of Research (WRAIR, Silver Spring, Maryland, USA). The lot of [16-^14^C] DHA (Lot No. 10839-113) used in this study and was synthesized by Research Triangle Institute (Research Triangle Park, North Carolina, USA). The stated specific activity and radiochemical purity was 21.83 mCi/mmol (77.3 μCi/mg) and 98.0%, respectively. Unlabeled DHA and radiolabeled compounds were stored at approximately -20°C.

### Animals

Male Sprague-Dawley rats obtained from Charles River Laboratories (Wilmington Massachusetts, USA) were used in this study. On arrival, the animals were acclimated for seven days (quarantine). The animals were housed individually and maintained in a room with a temperature range of 18–26°C (please give temperature in Celsius), 34–68% relative humidity, and 12-h light/dark cycles. Food and water were supplied *ad libitum *during quarantine and throughout the study. The animals were fed a standard rodent maintenance diet. The animal protocol was approved by Institutional Animal Care & Use Committee (IACUC), WRAIR, and the research was conducted in compliance with the Animal Welfare Act and other federal statutes. Regulations relating to animals and experiments involving animals adhered to principles stated in the Guide for the Care and Use of Laboratory Animals, NRC Publication, 1996 edition.

### Pharmacokinetic (PK) study

Traditional PK studies involve intermittent blood sampling and subsequent determination of drug concentrations in whole blood and plasma. The Culex automated blood sampler provides a way for blood withdrawal at preprogrammed intervals, which is more accurate and less labor intensive. Six rats in the PK group were implanted with a jugular vein catheter under an isofluorane anesthaesia. After the surgery, rats were allowed to recover for 96 hours with free access to food and water. The rats were dosed single i.v. at 3 mg/ml/kg with [^14^C] DHA (20 μCi/kg), which was prepared as a 25% cremophore/0.9% saline solution, through the tail vein. A 50–200 μl blood sample was withdrawn from the jugular vein catheter into a cooled vial, containing powdered heparin, at 5, 10, 30 minutes and 1, 1.5, 3, 6, 12, 24, 48, 72, 96, and 192 hours post-dosing according to a preset schedule. PK evaluation of [^14^C] DHA in plasma and whole blood, as well as unchanged DHA in plasma was conducted in individual rats.

### Tissue distribution of [^14^C] DHA study

Rats treated with a single [^14^C] DHA dose of 3 mg/20 μCi/kg intravenously (5 rats per group) were anesthaetized by isofluorane and euthanized via cardiac puncture at 1, 6, 24, 48, 72, 96, and 192 hours, post-dosing. Blood, liver, brain, eyes, adrenals, muscle, lungs, heart, liver, kidneys, spleen, stomach, small intestine, large intestine, perirenal fat and bone marrow samples were removed from each animal by gross dissection. The gastrointestinal tract (GI) was separated into the stomach (oesophagus and stomach), small intestine, and large intestine. Contents were procured from each GI segment. After flushing with saline, these and other tissues were stored. Tissues were rinsed gently, but thoroughly with water to remove remaining traces of blood before storage. Dissecting instruments were also washed between tissue procurements to avoid cross-contamination.

For most of the tissues 20% (w/w) aqueous homogenates of the tissue were prepared. Aqueous homogenates of the faecal and GI content samples were also prepared. Metabolism cages were washed with a solution of 2% Count-off^® ^and the volume was measured. Radioactivity in the plasma samples and in aliquots (approx. 0.02–1.0 ml) of urine and cage washings was determined. The blood cells (primarily erythrocytes), perirenal fat (approx. 0.2 g), bone marrow samples (approx. 0.1 ml), and approximately 300-μl aliquots of the homogenates of faeces, liver, kidneys, heart, brain, stomach and carcass were solubilized with SolvableTM tissue solubilizer (Perkin-Elmer Life and Analytical Sciences, Boston, MA). The homogenates of faeces, stomach content, and intestinal contents were extracted with 50% methanol for 24 hours. Aliquots of blood and pooled urine samples from selected intervals were also analysed for DHA content directly.

Total radioactivity in the collected tissue samples were quantified in duplicate. Weighed tissue aliquots (~0.2 g) were digested overnight in 2 ml of Solvable^® ^tissue solubilizer (Perkin-Elmer Life and Analytical Sciences, Boston, MA) at 50°C. Coloured samples were decolourized by adding a maximum of 0.2 ml of 30% H_2_O_2 _for 30–60 minutes. Scintillation cocktail (Hyonic Fluor; Perkin-Elmer Boston. Massachusetts, USA) was then added and radioactivity determined in a Perkin-Elmer Tri-Carb 3100 TR scintillation spectrophotometer (Packard Instrument Co., Downers Grove, Illinois, USA). Faeces and intestinal contents were thoroughly homogenized before taking aliquots. Plasma was also analysed using the HPLC-ECD system to measure the concentration of unchanged DHA, as described by Li *et al *[6].

### Mass balance and elimination of [^14^C] DHA study

Samples of urine and faeces were collected for a period of 192 hours after a single i.v. injection at 1, 8, 24, 48, 72, 96, 120, 144, 168, and 192 hours from individual stainless steel Nalgene metabolic cages (Nalgene Company, Rochester, New York, USA) designed for separate collection of urine and faeces. Metabolic cages were washed with a solution of 2% Count-off^® ^(Perkin-Elmer Life and Analytical Sciences, Boston, Massachusetts, USA) and the volume was measured. Duplicate portions (0.1 ml urine; 1 ml cage rinse) of each sample were radio-assayed after the addition of scintillation cocktail. The weights of individual faecal samples were obtained and the samples were subsequently homogenized in four volumes of de-ionized water. Quadruplicate portions (1.0 ml) of each homogenate were decolourized with 30% hydrogen peroxide, and digested with Solvable^® ^tissue solubilizer. After acidification with glacial acetic acid and addition of scintillation cocktail, samples were assayed for radioactivity. The samples were kept in at -20°C for two weeks prior to analysis.

### Metabolic profile of [^14^C] DHA study

About 2 ml of plasma and urine, and 1 – 2 g of faeces were used. Conjugates were hydrolyzed by glucuronidase (1000 FU/ml, Sigma-Aldrich, St. Louis, MO) at pH 4.6, 38°C for 24 hours. Active enzyme was checked by phenophthaleinglucuronide at the end of incubation. Urine and faecal samples were incubated for 48 hours and fresh enzyme was added after 24 hours. Total radioactivity in free, conjugated, and the remaining aqueous fractions were measured with liquid scintillation counters. Protein precipitates were dissolved in Solvable^R ^(200 mg in 1 ml) and counted for radioactivity after addition of scintillation cocktail.

### Protein binding of [^14^C] DHA study

Human blood samples (five men and five women) were freshly obtained from subject volunteers at the Department of Clinical Trials, WRAIR. Rat samples were freshly collected from five male and five female rats. Equilibrium dialysis was performed using Teflon dialysis chambers and Diachema standard membranes as described by Li and Hümpel [20]. The GD-4/90 Equilibrium Dialyzer (MM Developments, Ottawa) contained 20 micro-dialysis cells (2.0 ml/chamber). The molecular weight cutoff for dialysis membranes was 5000 D. Equilibrium was reached after four hours, when cells were rotated at 16 rpm and 37°C. Drugs were dissolved in plasma samples with various concentrations (0.15 – 57800 ng/ml) and all experiments were carried out for five hours.

### HPLC-ECD method

The analysis of cold DHA in plasma was performed according to a previously described HPLC method [21] with the following modifications: for simultaneous determination of artemisinin and DHA (artemisinin as an internal standard), the Agilent Eclipse XDB-C18 column 5 μm (4.6 × 150 mm) was used with an isocratic mobile phase consisting of 40% acetonitrile: 60% ESA Acid Metabolite A (70–4835) in the reductive mode (-400 mV) for the quantification of DHA. Validation and reproducibility were evaluated and a lower limit of quantitation (LLQ) of 2.31 ng/ml for DHA was determined. The inter- and intra-day coefficient of variation for accuracy and precision were all within ± 10%.

### Data evaluation

For pharmacokinetics, the concentration-time data of [^14^C] DHA in plasma and whole blood collected during the 192 hour treatment period was fitted to a three-compartment open model using a nonlinear, extended least-square fitting procedure (WinNonlin 5.2, Pharsight Corporation, Cary, NC) using weighted (1/concentration) nonlinear regression. The area under the curve (AUC) was determined by the linear trapezoidal rule with extrapolation to infinity based on the concentration of the last time point divided by the terminal rate constant. Extrapolations to time zero were done using zero concentration for intravenous dosing and using C_0 _values determined from the three-compartment model equation at time zero by i.v. route. Mean clearance rate (CL) was determined by dividing the dose by the AUC_inf _for i.v. injection. Mean residence time (MRT) was determined by dividing the area under the first moment curve (AUMC) by the AUC. The volume of distribution at steady state (Vss) was calculated as the product of CL and MRT. The concentration-time data of unchanged DHA was fitted to a two-compartment open model. Statistical analysis was conducted with Microsoft Excel using a Student's t-test for dependent samples to compare means of paired and unpaired samples between two groups.

## Results

### Dose formulation analysis

The results of the analysis of the i.v. formulation of [^14^C] DHA are designated at 3 mg/20 μCi/ml in this study. Chemical concentration and radioactivity analysis of samples taken from each dosing solution indicated the formulation was homogeneous. The mean concentration of DHA in the i.v. formulation was 2.87 to 3.12 mg/ml. The radioactivity content of the i.v. formulation was detected between 20.54 and 23.62 μCi/ml. The radiochemical purity of [^14^C] DHA was determined to be greater than 98% while cold DHA was greater than 99%.

### Pharmacokinetics of [^14^C] DHA and unchanged DHA

**Figure 1:**
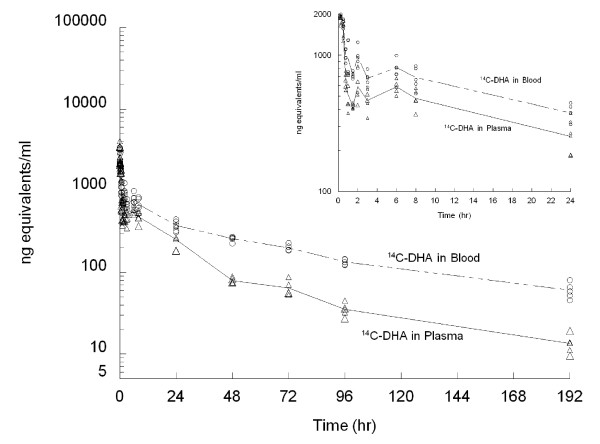
**Multiple peaks of mean concentration were shown in whole plasma (dash line with triangular markers) and in blood (solid line with circular markers) with the radioactivity (ng equivalents per ml), as well as PK profile of unchanged DHA in plasma (solid line) following a single 3 mg/20 μCi/kg intravenous injection of [^14^C]-dihydroartemisinin (14C-DHA) in rats *(n = 6)***.

Pharmacokinetic parameters calculated from mean levels of total radioactivity and unchanged DHA in either whole blood or plasma are shown in Table 1 after single i.v. administration of [^14^C] DHA. The concentration-time data of [^14^C] DHA in plasma and blood (Figure 1) collected during the 192 hours of the treatment period were fitted to a three-compartment open model. Radioactivity derived from [^14^C] DHA was eliminated from plasma in three phases with half-lives of 0.21, 15.49, and 75.57 hours, and from blood in three phases with half-lives of 0.18, 19.68, and 122.13 hours. Unchanged DHA was eliminated from the plasma in two apparent phases with half-lives of 0.25 and 1.03 hours (Table 1).

**Table 1:** Comparison of main pharmacokinetic parameters of [^14^C] dihydroartemisinin (DHA) in plasma and blood, as well as unchanged DHA in plasma at 3 mg/kg, in rats following a single intravenous dose

	14C-Dihydroartemisinin 3 mg/20 μCi/kg		Unchanged DHA 3 mg/kg
Parameters	Plasma Three-compartment	Blood Three-compartment	Plasma Two-compartment
C_max _(ng/ml)	3446 ± 396	3105 ± 430	1999 ± 507
T_max _(hr)	0.02	0.02	0.02
AUC_inf_. (ng*h/ml)	21014 ± 2718	52690 ± 4971	933.2 ± 125.4
t_1/2 _– alpha (hr)	0.21 ± 0.05	0.18 ± 0.02	0.25 ± 0.04
t_1/2 _– beta (hr)	15.49 ± 1.64	19.68 ± 3.88	1.03 ± 0.19
t_1/2 _– gamma (hr)	75.57 ± 7.34	122.13 ± 8.73	-
CL (ml/hr/kg)	144.63 ± 18.17	43.58 ± 30.70	3350.02 ± 423.37
Vss (ml/kg)	8216 ± 828	10792 ± 3771	1658 ± 540
MRT (hr)	57.00 ± 2.84	148.57 ± 90.24	0.49 ± 0.12
			
Cmax at first peak (ng/ml)	3446 ± 396 (0.02 h)	3105 ± 430 (0.02 h)	1999 ± 507 (0.02 h)
at second peak (ng/ml)	2373 ± 195 (0.19 h)	1557 ± 955 (0.94 h)	1638 ± 468 (0.38 h)
at third peak (ng/ml)	863 ± 537 (1.70 h)	978 ± 164 (2.00 h)	-
at fourth peak (ng/ml)	584 ± 65.5 (6.00 h)	833 ± 99 (6.00)	-

DHA = dihydroartemisinin; MRT = Mean residence time; MAT = Mean arrival time; - = no data is available. Mean ± SD (n = 5).

Peak concentrations (C_max_) of [^14^C] DHA and unchanged DHA in plasma at one minute were 3,446 ng equivalents/ml and 1,999 ng/ml, respectively. The corresponding AUCs of [^14^C] DHA and unchanged DHA were 21,014 and 933 ng equivalents·h/ml, suggesting DHA is extensively metabolized in animals. Similar to the AUC, the volume of distribution at steady state (V_ss_) was 8,216 ml/kg for radiolabeled drug and 1,658 ml/kg for unchanged DHA. Calculated clearance values (CL) for radioactivity and unchanged DHA in plasma were 144.63 and 3,350.02 ml/hr/kg, respectively (Table 1).

Following the i.v. administration of [^14^C] DHA four peaks of radioactivity were detected (Figure 1) within first 24 hours. The first peak concentration of radioactivity, 3,446 and 3,105 ng equivalents/g, was present in plasma and whole blood at one minute (earliest measured time point) after dosing. The second peak formed at 11 minutes with plasma at 2,373 ng equivalents/ml and at 56 minutes for whole blood with 1,558 ng equivalents/g. the third peak was seen at 1.7 hours with 863 ng equivalents/ml in plasma and at two hours with 978 ng equivalents/ml in blood. The fourth peak concentration of radioactivity appeared at 6 hours with 584 and 833 ng equivalents/ml in plasma and blood, respectively, revealing an enterohepatic circulation of [^14^C] in rats. Only two concentration peaks of unchanged DHA were found in plasma. The peak concentration of unchanged DHA was 1999 ng/ml at one minute and the second peak was 1638 ng/ml at 23 minutes after the intravenous injection.

### Tissue distribution of [^14^C] DHA

**Figure 2:**
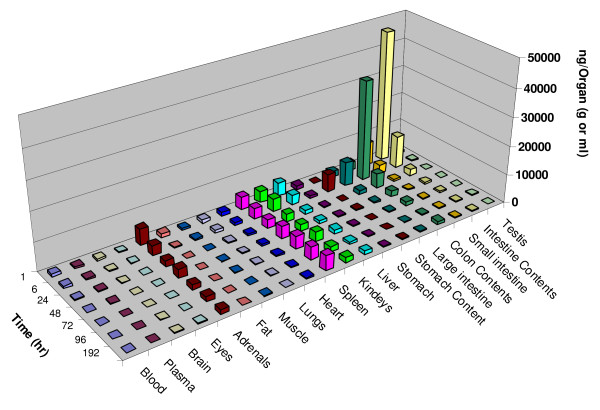
**Mean distribution profile of total radioactivity (ng equivalents per gram tissue) of dihydroartemisinin (DHA) in various tissues (column) at 1, 6, 24, 48, 72, 96, and 192 hr following a single 3 mg/20 μCi/kg intravenous injection of [^14^C] DHA in rats (*n *= *5*)**.

In tissues collected at 1 hr, approximately 62.6% of radioactivity in the total measured tissues was amassed in the small intestine and its content. Lesser amounts of radioactivity were distributed in other tissues (Table 2). The total amount of radioactivity in all measured tissues per gram was 87,187 ng equivalents. Six hours after the injection, high levels of radioactivity were still present in the intestine, colon, and in their contents with slow loss; however, drug distribution was significantly increased in other tissues such as kidney, spleen, liver, heart, adrenals, blood, muscle, colon and colon content (Figure 2). The total amount of radioactivity in all measured tissues per gram at six hours was similar to the 1 hr measurement at 86023 ng equivalents/g. The remarkable increases of radioactivity in other tissues at six hours may relate to redistribution from tissues, enterohepatic circulation, or the presence of DHA metabolites which have a smaller volume of distribution than DHA.

**Table 2:** Distribution (ng equivalents/tissue gram), half lives, and area under the curve (AUC) of [^14^C]-dihydroartemisinin in main organs and tissues of male rats following single intravenous dose at 3 mg/20 μCi/kg and animals were euthanized at 1, 6, 24, 48, 72, 96, and 192 hr after dosing (n = 5).

Parameters	1 hr	6 hr	24 hr	48 hr	72 hr	96 hr	192 hr	Half-lives (hr)	Tissue AUC (ng·hr/g)	% of total AUC
Blood	936.19	795.07	366.76	252.05	194.59	130.92	118.71	122.13 ± 8.73	45463 ± 6810	1.07
Plasma	591.34	540.83	216.03	93.02	64.70	34.58	22.84	75.57 ± 7.34	20750 ± 1031	0.49
Brain	578.68	326.57	160.11	127.38	138.35	119.67	133.52	72.21 ± 6.33	34189 ± 4885	0.81
Eyes	279.20	239.95	64.78	46.86	51.58	98.06	121.94	75.22 ± 9.40	16519 ± 3048	0.39
Adrenals	5614.75	3451.81	2034.62	3002.03	1630.57	1653.43	1543.60	92.48 ± 44.34	448390 ± 273502	10.59
Fat	1391.80	218.23	80.24	62.09	61.62	43.52	84.86	131.69 ± 21.94	25253 ± 1490	0.60
Muscle	698.14	499.29	171.74	163.19	174.04	92.20	115.14	64.45 ± 13.54	36371 ± 6994	0.86
Lungs	1161.72	1040.99	398.52	305.34	303.10	236.70	221.64	86.19 ± 18.00	85916 ± 12999	2.03
Heart	1512.27	1107.89	647.58	527.3	568.43	377.26	443.20	74.35 ± 25.84	104598 ± 24036	2.47
Spleen	4387.40	3684.97	3061.40	4216.03	4446.65	4464.57	5170.22	114.02 ± 57.67	617495 ± 410768	14.58
Kidneys	4097.87	4502.00	2535.56	2324.72	3286.27	1802.63	1819.89	77.90 ± 8.41	191781 ± 66422	4.53
Liver	4594.85	3516.69	1403.29	1104.94	1503.74	842.90	1270.24	95.18 ± 37.90	172474 ± 85780	4.07
Stomach	1366.22	932.33	464.21	342.30	337.48	229.69	218.27	72.58 ± 12.98	82125 ± 13428	1.94
Stomach Content	36.03	6289.79	22.45	98.12	18.69	11.76	343.37	16.75 ± 16.30	22362 ± 98089	0.53
Large intestine (LI)	1818.56	8394.43	1420.58	562.96	509.29	220.41	304.72	62.53 ± 43.59	70217 ± 25001	1.66
LI Content	2914.59	35567.01	5127.28	2037.53	1426.62	491.66	1333.83	37.20 ± 6.08	588542 ± 104261	13.90
Small intestine (SI)	8155.95	3014.58	821.84	335.42	389.72	235.61	304.12	49.99 ± 34.89	131264 ± 24631	3.10
SI Content	46426.85	11387.30	2283.61	633.61	575.05	176.57	250.03	28.73 ± 9.05	1492988 ± 63053	35.25
Testis	624.14	513.53	176.47	155.78	151.29	123.54	141.04	86.24 ± 40.68	48249 ± 6078	100.00
										
Total Amounts	87186.55	86023.26	21457.06	16390.68	15831.77	11385.67	13961.18	69.03 ± 22.13	4234952.00	1.14
% total recovery	34.57	34.10	8.51	6.50	6.28	4.51	5.53			

DHA = dihydroartemisinin; MRT = Mean residence time; MAT = Mean arrival time; - = no data is available. Mean

From 24 to 192 hours, measured radioactivity rapidly declined in all tissues except for the spleen (Figure 2). The total amount of radioactivity in all measured tissues per gram was 21,457, 16,390, 15,831, 11,385 and 13,961 ng equivalent at 24, 48, 72, 96, and 192 hours, respectively (Table 2). At 192 hours after dosing, residual activity (close to 5.53% of total amount collected) was still detected in some tissues and the highest levels of radioactivity were measured in spleen, kidney, adrenals, large intestine content, liver, and lungs. Radioactivity in the blood and plasma was measured in quantifiable levels at 192 hours after the single i.v. injection (Figure 1). Radioactivity in whole blood was always higher (1.8–4.6 fold) than in plasma throughout the period measured. Unchanged DHA was completely eliminated within 6 hr after 5–6 half-lives (Table 2), indicating that the long-lasting metabolites of DHA (> 192 hr) in the rats may be also related to slow release of DHA binding from RBC or other tissues (e.g. spleen) with subsequent metabolism and the enterohepatic circulation.

During the treatment period, measured levels of radioactivity were more than two-fold higher in the brain than in plasma; the AUC of radioactivity from 0 to 192 hours was 34,189 ng·h/g in the brain and 20,750 ng·h/ml in the plasma. Excepting small intestine (35.25%), the highest levels of the radioactivity were detected in the spleen. The measured amount was 4,387 ng equivalents/g at 1 hr after dosing. From 6 to 24 hours, the amount decreased from 3,685 to 3,061 ng equivalents/g (Table 2). However, the amount of radioactivity increased again to 4,216 ng equivalents/g by 48 hours; thereafter, the level constantly increased up to 5,170 ng equivalents/g at 192 hours, with was the highest AUC (617,495 ng·h/g) and the highest percentage (14.58%) of total amount of radioactivity in spleen, when compared to other tissue. In other measured tissues, the highest to lowest AUCs were found as the follows: large intestine content (588,542 ng·h/g), adrenals (448,390 ng·h/g), kidneys (191,781 ng·h/g), liver (172,474 ng·h/g), small intestine (131,264 ng·h/g), heart (104,598 ng·h/g), lung (85,916 ng·h/g), and large intestine (70,217 ng·h/g) (Table 2).

The longest half-life of radioactivity was measured in the fat at 131.69 hours, followed by the blood (122.13 h), spleen (114.02 h), liver (95.18 h), adrenals (92.48 h), testis (96.24 h), lungs (86.19 h), and so on. In contrast, the radioactivity lasted much less in the contents of stomach, small intestine, and large intestine with half-lives of 16.75, 28.73, and 37.20 hours, respectively.

### Mass balance determination

The total recovery of radioactivity in excreta (urine and faeces) and cage rinses collected from animals through 192 hours after single i.v. administration is shown in Table 3. Through 192 hours after injection, approximately 52.09%, 40.39% and 1.88% of the dose was eliminated in urine, faeces, and cage rinses, respectively. The urinary excretion was the major route of elimination, accounting for 44–61% of the administered dose. Faecal excretion was the secondary route of elimination, accounting for 27–53% of the administered dose. The recovery of [^14^C] DHA from urine, faeces and cage washing solution was 94.36% of total dose during the 9 days of collection.

**Table 3:** Percentages of renal and fecal excretion of total radiolabel following single intravenous administration of [^14^C] dihydroartemisinin with 25% cremophore EL/0.9% saline formulation at 3 mg/kg to rats (n = 5).

Sample time (days)	Urine	Feces	Cage wash
0–0.04	4.18 ± 1.06		
0.04–0.33	25.49 ± 1.98	1.52 ± 0.58	
0.33–1.0	14.01 ± 5.11	29.56 ± 11.75	
1–2	4.78 ± 1.30	6.14 ± 1.71	
2–3	1.63 ± 0.92	1.64 ± 0.78	
3–4	0.72 ± 0.19	0.78 ± 0.31	
4–5	0.57 ± 0.25	0.46 ± 0.09	
5–6	0.46 ± 0.37	0.15 ± 0.06	
6–7	0.16 ± 0.02	0.11 ± 0.05	
7–8	0.09 ± 0.04	0.04 ± 0.04	
Total	52.09 ± 8.94	40.39 ± 13.22	1.88 ± 2.27
			
Ratio of excretion (urine/feces)	1.37 ± 0.39		
Balance (% of dose)	94.36 ± 16.71		
			
Elimination parameters			
Peak height (μg/rat)	234.48 ± 33.74	292.39 ± 150.29	
Peak time (days)	0.37 ± 0.06	0.67 ± 0.09	
Fast elimination phase (days)	0.32 ± 0.07	0.35 ± 0.11	
Slow elimination phase (days)	1.36 ± 0.25	1.29 ± 0.47	

All values expressed as % of dose, mean ± SD, n = 5.

### Urinary and faecal elimination

**Figure 3:**
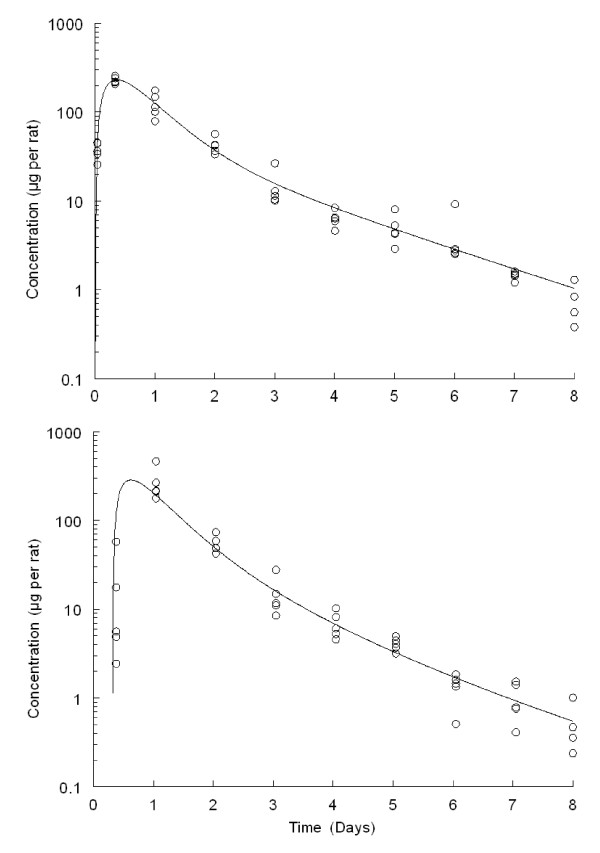
**Individual elimination profile of total radioactivity (μg equivalents per rat) of dihydroartemisinin (DHA, circular markers) in urine (top) and faeces (bottom) and computer fitted curves (solid line) by pharmacokinetic parameters following a single 3 mg/20 μCi/kg intravenous injection of [^14^C] DHA in rats during 8 days collection (*n *= *5*)**.

The elimination parameter estimates after intravenous dosing of [^14^C] DHA at 3 mg/kg are presented in Table 3. Results showed the greater than 74.76% of the total dose was excreted within first 24 hours. The maximum [^14^C] radioactivity concentration in urine was observed with 243.48 μg or equivalents per rat at 0.37 days. The elimination of [^14^C] label from urine occurred in two phases, fast and slow, with half-lives of 0.32 and 1.36 days (Figure 3, top). Similar to urine, fast and slow elimination phases were also exhibited in faeces. A peak concentration of 292.39 μg equivalents per rat was seen in faecal matter at 0.67 days after dosing. The half-lives of [^14^C] radioactivity were 0.35 and 1.29 days for fast and slow phases in faeces, respectively. The elimination of radioactivity in faeces was not immediately like urine and was delayed with a lag time of 0.31 days (Figure 3, bottom), while the lag time for urine was < 0.02 days.

### Metabolite profiles of [^14^C] DHA

Unchanged DHA was rapidly and completely eliminated from rats within 6 hours following single i.v. administration. The total radioactivity (metabolites) lasted longer than 192 hr in plasma, urine and faeces. Conjugation studies showed drug conjugation of the dose in plasma and urine (up to 89% and 95%, respectively) is a major metabolic pathway of [^14^C] DHA, and that conjugation is time-dependent in rats (Table 4). Metabolites of DHA were quantified before and after hydrolysis with beta-glucuronidase to give free and conjugated fractions of plasma, urine and faeces. In the beginning, within five minutes after dosing, the drug total conjugation fractions were similar to free fraction with the ratio of conjugation to free from 1.07 in plasma. The ratio was increased to 1.86 at 10 minutes and then rapidly increased to 4.48 at 1 hour after dosing. After dosing, the ratio moderately elevated up to 15.1 at 192 hours (Table 4).

**Table 4:** Distribution, concentration and half-lives of [^14^C] dihydroartemisinin (DHA) in total, free, glucuronide and other conjugation fractions in plasma or urine following a single intravenous dose at 3 mg/20 μCi/kg in rats.

Time	Total	Free fraction	Glucuronide conjugation	Other conjugation fraction
Plasma (n = 5)				
0	0.00	0.00	0.00	0.00
0.017	3446.38 ± 396.35	2253.63 ± 537.31	403.87 ± 124.77	583.17 ± 155.52
0.083	2279.43 ± 213.77	925.14 ± 226.81	413.56 ± 69.46	589.70 ± 102.30
0.167	2090.86 ± 180.56	697.05 ± 88.04	480.62 ± 49.17	818.11 ± 88.12
0.25	1987.48 ± 378.69	685.05 ± 193.99	360.93 ± 65.83	684.75 ± 161.05
0.50	1489.58 ± 231.59	461.20 ± 56.83	305.81 ± 26.23	627.62 ± 59.19
0.75	654.49 ± 87.56	157.51 ± 28.11	131.60 ± 29.09	341.12 ± 66.12
1.0	514.20 ± 139.37	105.36 ± 55.10	122.76 ± 36.01	348.30 ± 125.91
1.5	419.48 ± 17.06	75.83 ± 9.85	74.17 ± 11.64	268.16 ± 14.76
2	589.26 ± 154.02	90.58 ± 33.58	110.13 ± 44.55	407.78 ± 112.11
3	466.65 ± 82.13	74.40 ± 16.25	80.73 ± 14.20	321.61 ± 69.08
6	584.41 ± 65.49	51.49 ± 15.64	49.34 ± 22.19	363.63 ± 162.04
8	481.59 ± 79.35	49.44 ± 13.34	57.72 ± 13.56	388.11 ± 72.73
24	253.62 ± 78.47	14.46 ± 3.15	20.04 ± 5.05	188.12 ± 47.66
48	79.17 ± 6.71	7.41 ± 3.24	8.55 ± 1.68	77.07 ± 14.36
72	64.38 ± 14.66	4.89 ± 1.83	4.31 ± 1.19	48.23 ± 12.74
96	35.26 ± 6.54	2.09 ± 1.39	2.25 ± 0.87	25.39 ± 7.32
192	13.48 ± 3.65	1.01 ± 0.92	0.76 ± 0.72	14.49 ± 1.62
				
AUC (ng·h/ml)	21708 ± 2695	2188.2 ± 286.4	2177.6 ± 299.6	14193.6 ± 6838.7
t_1/2 _(hr)	15.49 ± 1.64	4.01 ± 0.99	6.66 ± 1.04	20.25 ± 1.32
% of total	100.2 ± 12.68	10.08 ± 1.32	10.03 ± 1.38	79.14 ± 8.22
				
Urine (n = 5)				
0	0.00	0.00	0.00	0.00
0.04	37186.4 ± 8200.9	2253.63 ± 113.44	1611.28 ± 756.20	21546.88 ± 8219.11
0.33	228363.9 ± 20101.9	2693.12 ± 1293.63	8359.94 ± 1428.22	39881.66 ± 5139.02
1.0	123776.8 ± 38621.3	535.48 ± 339.64	732.58 ± 373.98	10784.80 ± 6618.73
2.0	42340.5 ± 8839.2	184.08 ± 70.94	97.20 ± 50.00	2003.77 ± 1218.99
3.0	14248.3 ± 6958.5	86.77 ± 56.02	29.60 ± 15.80	655.31 ± 605.27
4.0	6355.5 ± 1351.3	43.20 ± 26.91	17.54 ± 11.47	278.99 ± 208.65
5.0	4984.6 ± 1922.7	35.21 ± 39.14	12.70 ± 9.90	310.60 ± 306.84
6.0	4006.4 ± 2925.2	22.16 ± 28.94	7.77 ± 3.88	289.69 ± 338.11
7.0	1451.9 ± 149.2	9.75 ± 4.98	6.21 ± 2.92	147.26 ± 104.46
8.0	780.1 ± 344.4	5.77 ± 3.86	3.28 ± 1.66	91.76 ± 59.41
				
AUC (ng·h/ml)	43624 ± 10214	2213.6 ± 933.4	5069.3 ± 928.2	35657.7 ± 9342.1
t_1/2 _(day)	1.36 ± 0.25	1.25 ± 0.11	1.30 ± 0.16	1.64 ± 0.11
% of total	98.43 ± 23.38	5.11 ± 1.45	12.22 ± 3.14	82.67 ± 3.96

AUC = area under curve; t_1/2 _= half-life

### Protein binding of [^14^C] DHA with rat and human plasma

**Figure 4:**
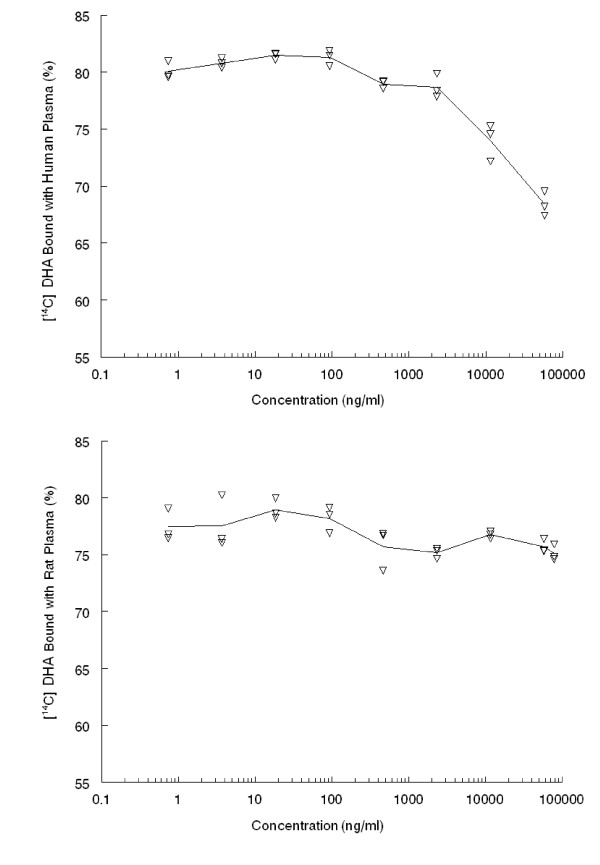
**Individual protein binding of ^14^C-DHA in male human (top) and male rat (bottom) plasma (triangle markers) and mean value in the human plasma (solid line) after incubation in variable concentrations of ^14^C-DHA (0.15, 0.74, 3.7, 18.5, 92.5, 462, 2312, 11560, and 57800 ng/ml) at 37°C for 5 hours (*n *= *3*)**.

[^14^C] DHA revealed a higher binding percentage with human and rat plasma proteins (76–82%) when incubated with undiluted plasma samples at concentrations of 0.15 to 57800 ng/ml at 37°C for five hours. There was a concentration dependent decrease in the binding of [^14^C] DHA. At higher concentrations (> 25 ng/ml) the binding percentage declined from 82% to 66%, indicating that the maximum binding percentage occurred in the concentration range of 0.15 – 462 ng/ml (Figure 4, top). The binding capacity of DHA in rat plasma was not significantly different with drug concentration vary (Figure 4, bottom).

## Discussion

Previous studies have established the role of biliary excretion in the elimination of artemisinin metabolites from rats [18,22,23] and mice [24]. In the present studies, the tissue distribution and metabolic profiles of DHA by tissue dissection techniques (TDT) method confirmed the results from the published literature. Findings from this study provide evidence of the extent of metabolism of DHA in rats. The outcomes of this study indicate very little unchanged DHA is eliminated by direct excretion from kidneys (<1% of dose); rather most (99%) occurs via metabolism (hydrolysis and oxidation). Data presented in this study allows a comprehensive characterization of DHA with pharmacokinetics, tissue distribution and its metabolic profiles.

Unchanged DHA was eliminated from plasma with a terminal half-life of 1.03 hours after i.v. administration. The Vss of unchanged DHA was estimated to be 1,658 ml/kg, which was a half of Vss of unchanged artesunate (3982 m/.kg) in rats published previously [18], indicating that DHA has a relatively small volume of distribution and the compound was distributed to total body water. The calculated plasma clearance of DHA was 3,350 ml/hr/kg, which is less to rat normal hepatic blood flow (4,800 ml/hr/kg). This is probably related to uptake and binding of DHA in red blood cells. The results indicate even though the distribution of unchanged DHA is apparently limited to total body water, the radioactivity derived from [^14^C] DHA was extensively distributed throughout rats and lasted for an extended period (at least 192 hours). The average ratio of blood to plasma per ml was 2.18 during the 192 hr period. High concentrations of [^14^C] DHA present in RBC is very important for the anti-malarial agent in blood stage treatment. The drug concentration in RBC is about 3.54 times higher than that in plasma by calculated using a haematocrit of 0.48, indicating that the powerful anti-malarial potency of DHA in animal species and humans may relate to the high drug levels in the RBC.

An understanding of artemisinin-associated neurotoxicity *in vivo *requires knowledge of the penetration of the cerebrospinal fluid (CSF) by these drugs. Such data is sparse. Artemisinin derivatives cross the blood-brain barrier in rats [24]. Artesunate is converted stoichiometrically to DHA, which is highly lipid soluble and has a low molecular mass (284 Da), favouring penetration of CSF [25]. Since DHA has relatively low solubility in water, it should be able to cross cell membranes and be conjugated. After DHA treatments in patients, no unchanged DHA was detected in CSF but [^14^C] DHA levels in CSF increased with time while DHA levels in plasma fell, suggesting continued influx, with a slower efflux of DHA. [^14^C] DHA may accumulate in CSF during frequent DHA dosing [25]. In the present study, the radioactivity in brain tissues (AUC: 34,189 ng·h/g) was more than twice as high as in plasma (AUC: 20750 ng·h/ml). The half-life of [^14^C] DHA in brain tissue was 72.21 hr, which was also longer than that in plasma, 63.04 hr. The results indicated that the residence time of [^14^C] DHA is longer in the brain than that in plasma. This may reflect a sink effect of DHA and/or other metabolites and an uptake transfer by lipid-rich brain structures [25]. The presence of DHA metabolites in brain tissue, and any association with neurotoxicity is currently unknown.

The current study demonstrates DHA undergoes rapid metabolism via conjugation and biliary elimination. The results of the tissue distribution studies show 53–86% of total radioactivity to be found in the content of small intestine within the first hour after i.v. dosing. At one hour after dosing most of the [^14^C] was present in the liver, where it was excreted into the intestines via bile ductules; this suggests the higher hepatic biotransformation of DHA is similar to metabolic profile of artesunate and other artemisinin compounds [18,22,23]. Urinary and faecal excretion data obtained after i.v. administration of [^14^C] DHA demonstrated that approximately 52% of the dose was eliminated in urine and 40% in faeces within 192 hours after dosing. The finding also suggests a majority of [^14^C] DHA may be first excreted in bile and re-absorbed from intestines.

The multiple radioactivity peaks shown in the plasma, blood and various tissues indicate the presence of enterohepatic circulation of [^14^C] DHA in all animals. The drug plasma concentration profile revealed that [^14^C] DHA formed a second, third and fourth peak at 11 minutes, 1.7 h and 6 h, respectively, after i.v. injection (Figure 1). This again suggests that the multiple increases in drug concentration are due to either enterohepatic circulation or the presence of a multiple absorption phases [26-28]. Usually, the multiple peaks in oral concentration-time profiles, following drug administration, are a result of: discontinuous absorption along the gastrointestinal tract, post-absorptive storage and release, and/or enterohepatic circulation [29]. Therefore, the multiple peaks in the present study and the high amounts of radioactivity in biliary excretions after the i.v. injection of DHA strongly suggest the presence of enterohepatic circulation [30-33].

## Conclusion

The binding capacities of [^14^C] DHA with human and rat plasma at higher percentages, 76–82%, are seen in a concentration dependent manner. Radioactivity DHA showed very longer half-lives of 75.57 and 122.13 hr in plasma and blood, respectively when compared to unchanged DHA with only 1.03 hr, indicating long-lasting metabolites presented in this study. The drug clearance and distribution volume were 43.58–144.63 ml/hr/kg and 8216–10792 ml/kg, respectively, for total radioactivity, but were 3350 ml/hr/kg and 1658 ml/kg, respectively, for unchanged DHA. High tissue distribution was found in rat spleen, followed by kidneys, adrenals, liver, during the 192 hr period. In rat brain, the total concentration of [^14^C] was 2-fold higher than that in plasma, indicating the radioactivity could easily penetrate the brain-blood barrier. Total radioactivity distributed in RBC was about three- to four-fold higher than that in plasma, suggesting that the powerful anti-malarial potency of DHA in treatment of blood stage malaria may relate to the high RBC binding. Biliary excretion and multiple concentration peaks of DHA has been demonstrated with high excretion via bile and approximately 89–95% dose of conjugations in blood, urine and faeces. However, the majority of elimination of [^14^C] DHA is through urinary excretion (52% dose) due to a most likely drug re-absorption in the intestines and enterohepatic circulation, as suggested by the biliary metabolism and the multiple concentration peaks in blood, plasma and tissues. The long-lasting metabolites of DHA (> 192 hr) in the rats may be also related to slow release of DHA binding from RBC or other tissues (e.g. spleen) with subsequent metabolism and the enterohepatic circulation.

## Competing interests

The authors declare that they have no competing interests.

## Authors' contributions

LX, JZ and QL performed all experiments. LX contributed to the experiment design, coordination, supervision and study performance of the research work, data entry, cleaning and analysis, and paper writing. QL contributed to the study design, protocol and final report writing, radiation work administration, data statistical analysis and review of the paper. JZ contributed to animal study, bench works and data management and analysis. PW contributed to study supervision, data analysis, report review and interpretation as well as manuscript review. All authors participated in the interpretation, and writing of the paper and read and approved the final manuscript.

## Acknowledgements

This study was supported by the United States Army Research and Materiel Command. The opinions or assertions contained herein are the private views of the authors and are not to be construed as official, or as reflecting true views of the Department of the Army or the Department of Defense.
